# Does packed red cell transfusion provide symptomatic benefits to cancer palliative patients?: a longitudinal study from a single private oncology center in Nepal

**DOI:** 10.1186/s12904-019-0454-1

**Published:** 2019-08-06

**Authors:** Sameer Timilsina, Sirisa Karki, Santosh Timalsina, Aajeevan Gautam, Sabitri Sharma

**Affiliations:** 1 Department of Physiology, Tribhuvan University, Chitwan Medical College, Post Box No.: 42, Bharatpur-5, Chitwan, Nepal; 2 Department of Pharmacology, Tribhuvan University, Chitwan Medical College, Bharatpur-5, Chitwan, Nepal; 3 Department of Biochemistry, Tribhuvan University, Chitwan Medical College, Bharatpur-5, Chitwan, Nepal; 4 Department of Anatomy, Tribhuvan University, Chitwan Medical College, Bharatpur-5, Chitwan, Nepal; 5 Palliative Care Unit, Nepal Cancer Hospital and Research Center, Harisiddhi, Lalitpur, Nepal

**Keywords:** Fatigue, Dyspnea, Hemoglobin, Packed red cell, Transfusion, Palliative care

## Abstract

**Background:**

Palliative patients generally present with symptoms of dyspnea, easy fatigability, lethargy and feeling of being unwell which can broadly be attributed to one root cause: cancer-related anemia. So, packed red cell transfusion is often carried out aiming to improve patients’ functional status. Different cut off hemoglobin values have been suggested, with Hb < 9 g/dL the most commonly accepted. The present study aims at evaluating and comparing the benefits in subjective symptoms of fatigue and breathlessness among transfused and non-transfused palliative patients on Day 0 and Day 7.

**Methods:**

Hemoglobin values, anemia related subjective symptoms of fatigue and breathlessness were recorded from 122 patients. The patients were re-evaluated on day-7 post-transfusion. The pre and post-transfusion symptomatic benefit was compared in both transfused and non-transfused palliative care patients.

**Results:**

The currently practiced hemoglobin trigger for packed red cell transfusion is 10 g/dL. The units of packed red cell to be transfused was decided according to the hemoglobin values targeting the rise to > 10 g/dL. A mean 1.36 units were transfused. Statistically significant improvement was observed in patient reported symptoms of fatigue and breathlessness among both transfused and non-transfused palliative patients.

**Conclusion:**

Anemic cancer palliative patients were found to benefit following packed red cell transfusion, suggesting a favorable association between the transfusion and patient-reported fatigue and dyspnea.

## Background

The global burden of cancer is on the rise in the middle and low economic countries accounting for 82% of total cancer population and 70% of cancer related deaths worldwide [1, 2]. The rise has been attributed to improved management of communicable diseases, thereby, leading to increasing aging population and incidence of non-communicable diseases. Besides this, rapid urbanization has further assisted in escalating prevalence of established risk factors like physical inactivity, obesity, pollution and changing reproductive patterns [1]. World Health Organization (WHO) defines Palliative care as “an approach that improves the quality of life of patients and their families facing the problem associated with life-threatening illness, through the prevention and relief of suffering by means of early identification and impeccable assessment and treatment of pain and other problems, physical, psychosocial and spiritual” [3]. In spite of massive demands of palliation and hospices, palliative care physicians and centers are few and far between in our country [4].

Cancer palliative patients present with a number of symptoms secondary to the primary disease including pain, dyspnea, lethargy, easy fatigability and personal feeling of being unwell. Anemia is the most frequently encountered clinical finding needing prudent attention in cancer palliative patients, believed to cause a majority of symptoms. In cancer, several cytokines namely tumor necrosis factor -α (TNF-α), transforming growth factor -β (TGF-β), interleukin-1 (IL-1), IL-6 and interferon-γ interfere normal erythrocyte production by modulating iron metabolism and blunting erythropoietin effect thereby causing anemia [5]. Cancer Induced Anemia (CIA) [6] is associated with a decline in patients performance status, cognitive function, decreased quality of life (QoL) and overall survival, therefore, correction of anemia is likely to improve survival benefits [7]. Anemia can be treated using blood transfusions, iron and vitamin B12 supplementation and use of erythropoiesis-stimulating agents (ESA) [8]. Correction of anemia in palliative settings using blood transfusion could be beneficial as it has rapid response to anemia induced symptoms. However, this does not mean the treatment of anemia should only be restricted to blood transfusion. As blood products are scarce resources it should be used vigilantly and dogmatically. Studies on beneficial effects of transfusion in cancer palliative patients are highly inconclusive, and blood transfusion is highly individualized [9]. Rather than being dictated by hemoglobin (Hb) values, transfusion of blood products should be aimed at improving the functional status and QoL [10–13]. Hemoglobin is the most widely accepted standard transfusion trigger in all anemic patients and is usually based on subjective symptoms of easy fatigability, shortness of breath and lethargy [14]. In absence of any cardiac disease, the recommended hemoglobin value for restricted packed red cell transfusion is 7-9 g/dL and is 10-12 g/dL for liberal transfusion [15]. No standard guidelines can be found in the literature regarding transfusion in palliative patients. Some studies have shown beneficial effects of transfusion immediately after transfusion but wearing off after 2 weeks, while other studies fail to report any benefits [16, 17].

Cancer Related Fatigue (CRF) is a subjective feeling of persistent distress, tiredness, or exhaustion related to cancer or cancer related treatments that would otherwise not interfere with normal functioning [18]. CRF is multifactorial with pro-inflammatory cytokines, IL-1, metabolic and insulin insensitivity as most commonly proposed causes [19]. It has been reported to be as high as 90% in cancer patients. Anemia is the most common reversible cause of CRF in cancer palliative patients and correction of anemia can improve the symptoms of fatigue. The current study aims at evaluating the changes in subjective symptoms of fatigue and breathlessness in transfused and non-transfused cancer palliative patients.

## Methods

This was a hospital-based longitudinal study conducted from July to December 2017 at Nepal Cancer Hospital and Research Center, Harishiddi, Lalitpur (1400 m above sea level). Ethical clearance for the study was obtained from IRC-NCHRC. The study included a total of 122 cancer palliative patients both admitted to the hospital and attending palliative OPD. Information about the type of cancer, stage and site was not recorded. A written consent was obtained from all the patients for participation in the study. Patients with advanced cancer on diagnosis (Stage IIIb and IV), progressive and refractory or recurrent to chemotherapy and those who did not accept any form of active-cancer treatment either due to religious, social or economic reasons were included in the study. Patients receiving active cancer treatment; chemotherapy or radiotherapy and those with hematological malignancies were not included in the study. Patients with hematological malignancies both actively receiving chemotherapy and those in remission followed up with either oncologists or hematologists, so these groups of patients were not included in the present study. Also, patients who had have transfusions within the past 3 months and those who received ESA within the past 2 months were also excluded from the study. Participants were evaluated for subjective symptoms of fatigue and breathlessness. Fatigue was categorized with the subjective response as “yes” or “no” answers and evaluation of breathlessness was done using the Dyspnea Index (DI) and was categorized as very severe (0) to no breathlessness (4). Hemoglobin values were obtained from the laboratory using automatic hematology analyzers. Appropriate internal quality controls were run before assay of the sample. Packed red cell (350 ml) transfusion was ordered for hemoglobin values less than 10 g/dL and subjective symptom of fatigue and breathlessness. Every unit of packed red cell (350 ml) transfused was expected to raise the hemoglobin levels by 1 g/dL and follow-up hemoglobin level was targeted at a value above 10 g/dL. Both the group of patients (transfused vs. non-transfused) were reevaluated on day 7 and reassessed for subjective symptoms of fatigue and breathlessness and the response was noted accordingly. Statistical analysis was done using the program SPSS version 20. Hemoglobin values less than 10 g/dL was considered as anemic. The numerical values were expressed as mean ± SD and categorical variables as percentage. McNemar’s test was done to evaluate the difference in fatigue and dyspnea score pre and post-transfusion.

## Results

A total of 136 patients attended the palliative care ward and OPD during the course of the study. Six of them did not agree to participate in the study due to social or religious reasons, 5 of them did not fit the inclusion criteria and 3 of them could not be followed up (1 passed away and 2 were unreachable). Of the remaining 122 patients in the study, 63(51.6%) were males and 59(48.4%) were females. The median age was 60 years (range 13–88 years). The mean age was 55.32 ± 16.79 years with 61.5% (75/122) of patients above 50 years.

The mean hemoglobin concentration was 10.53 ± 1.51 g/dL. Of the 122 patients in the study 44(36.1%) were anemic and 78(63.9%) of the patients were non-anemic. Table 1 shows the mean hemoglobin concentration of anemic and non-anemic participants in the study along with number of anemic and non-anemic patients with symptoms of fatigue and breathlessness.

**Table 1:** Variables in anemic and non-anemic study group

	Anemic	Non-anemic
No of patients	44 (36.1%)	78(63.9%)
Mean Hb g/dL	9.03 ± 0.72	11.38 ± 1.14
Fatigue	36/44	34/78
Breathlessness	32/44	3/78

All 44 anemic patients with Hb < 10 g/dL were transfused packed red blood cell irrespective of symptoms. Also, 2 patients with Hb > 10 g/dL but with symptoms of breathlessness underwent transfusion. A total of 46 patients received 64 pints of packed red cell transfusion during the course of the study with an average transfusion rate of 1.36 ± 0.67 pints. The lowest hemoglobin level for which packed cell was transfused was 7.1 g/dL and the highest was 10.4 g//dL. Of the total transfusion, 32 of the patients received 1 pint, 10 of them received 2 pints and 4 of them received 3 pints of packed red cell transfusion. The primary transfusion indication was low hemoglobin concentration and/or symptoms of fatigue or breathlessness.

Table 2 compares the symptoms of breathlessness and fatigue among transfused and non-transfused palliative patients.

**Table 2:** Comparison of fatigue and breathlessness symptoms among transfused (2A) and non-transfused patients (2B) pre and post transfusion

2A	Packed cell Transfusion Status		*p* value
*n* = 46	Pre-Transfusion	Post-Transfusion	
Fatigue	37 (80.4%)	20 (43.4%)	< 0.001
Breathlessness	35 (76.0%)	9 (19.5%)	< 0.001
2B *n* = 76	Packed cell not transfused		p value
	Before	After	
Fatigue	33 (43.4%)	22 (28.9%)	< 0.05
Breathlessness	0	0	–

70 (57.4%) of the total patients presented with complaints of fatigue and 35 (28.7%) with breathlessness. Prior to transfusion, 80.4% (37/46) patients had fatigue, but only 43.4% of them complained of fatigue post-transfusion. Likewise, improvement in breathlessness symptom was noted in 56.4% of patients post-transfusion. Symptom relief was found to be statistically significant in packed cell transfused patients (*p* < 0.001).

In patients who did not receive transfusion, there was a 14.5% reduction in fatigue on follow up after 7 days. The improvement in fatigue score was also statistically significant in non-transfused group (*p* < 0.05). Among the non-transfused group of patients, none of the patients had breathlessness on presentation at both times.

Early transfusion reactions like thrombophlebitis 2.1% (1/46) and non-hemolytic febrile reactions 10.8% (5/46) was noted.

## Discussion

Anemia in palliative patients can be a result of global impact of cancer on bone marrow which can occur even before any anticancer treatment. Cytokines like TNF-α blunts erythropoietin effect and IL-1, IL-6 and interferon-γ interfere with iron metabolism causing anemia. Chronic inflammation, malnutrition, bleeding, renal insufficiency, anemia of chronic disease and other co-morbidities have been attributed as a cause of anemia in cancer patients [20]. Anemia in cancer palliative patients in the present study was estimated at 36.1% similar to Ludwig H. et al. [21] while others have reported prevalence as high as 65% [20]. Maccio A. et al. [20] reported a higher percentage of CIA in advanced cases and compromised performance status (PS) but the present study did not categorize the severity of cases and PS.

Blood transfusion is useful in the management of anemia in palliative patients, especially when anemia in cancer patients decreases the functional status and QoL. The transfusion rate was 37.7% which was slightly higher than previous studies [8, 21–23]. Moreover, several studies have observed that palliative patients at the end of their life are more likely to receive additional transfusions than normal setting [23–25].

The currently practiced hemoglobin trigger for packed cell transfusion was < 10 g/dL, atypical of the internationally recommended value of < 9 g/dL [8, 26], nonetheless subgroups of breast cancer patients have shown survival benefits when transfused at Hb < 10.5 [27]. Due to the lack of expert palliative care manpower in the country, practicing oncologists are added with extra burden working as palliative care physicians. Oncologists’ priority of chemotherapy over palliative care and a lack of adequate palliative care expertise could yet be another factor influencing transfusion rates. Also, liberal transfusion of Hb < 10 g/dL, over restrictive transfusion of Hb < 7gdL was practiced in the institute. However, no studies have yet been conducted as to identify which groups of patients are likely to benefit most from transfusion [16, 28]. In normal 70 kg adult, one unit of packed red blood cells should increase the levels of hemoglobin by 1 g/dL and hematocrit by 3% [29]. We believe that altitude has nothing to do with the liberal transfusion strategy currently adopted at this center.

In spite of blood being a scarce resource, transfusion was considered as an alternative over ESA owing to some disadvantages including cost, 4–8 weeks latent period before observing maximum benefit and NCCN guidelines suggesting using ESA only in chemotherapy induced anemia [7, 26, 30]. Attending physicians, patients’ and their family alike, seeking immediate symptomatic improvements in order to avoid lengthier hospital stay to minimize cost, could yet be another reason for practicing blood transfusion over ESA.

In the present study, the mean hemoglobin concentration in anemic cancer palliative patients was found to be 9.03 ± 0.72 g/dL which was again higher than previous studies as hemoglobin cutoff values for transfusion was higher in our study [8, 16, 22, 23]. Likewise, the average pints transfused in the study was 1.36 ± 0.67, higher than reported in previous studies [8, 16]. However, 32/46 (69.5%) of patients were transfused with only 1 pint of packed cell which was similar to referenced literatures [8, 16]. This can again be explained by the low cutoff hemoglobin values.

Mercadante S. et al. reported short-term improvement in fatigue and dyspnea symptoms similar to present study [31]. Hemoglobin levels above 12 g/dL have shown to improve cancer related fatigue and overall QoL [32, 33]. Studies have advised appropriate use of packed red cell to treat fatigue symptom rather than treating anemia [34].Thirty seven percent patients reported improvement in fatigue symptoms similar to Brown E. et al. and Preston NJ. et al. [16, 33] and 56.4% in breathlessness symptoms. Statistically significant symptomatic improvement of fatigue and breathlessness was observed in both categories of patients (transfused vs. non-transfused). As the post-transfusion data was collected on day 7, the beneficial effect of transfusion beyond the seventh day is inconclusive. In transfused patients, the improvement could have been due to correction of CRF secondary to anemia. Likewise, in patients who were not transfused, the improvement could have been due to medical management and bed rest. Also, tendency of a person seeking attention when sick and hopefulness of being cured on visiting the clinician could be an additional reason for both group of patients. As it is commonly believed, a clinician even on touching or talking to a patient is able to cure 90% of the disease symptoms. This self-psychology of being cured upon consulting a physician could also be a factor of improvement of symptoms in both sets of patients. The present study is not in a condition to postulate the reasons behind the improvement, but one thing that has been demonstrated is the association between packed red cell transfusion and patient-reported fatigue and dyspnea. Previous study by To TH. et al. [35] using objective-scale measures have failed to show transfusion benefits correlating with subjective responses, though subjective improvement was reported.

Goksu SS et al. [36] showed survival benefits in palliative patients following transfusion but we did not include survival benefits, nutritional and socioeconomic status as a part of the study.

In the present study, we speculated anemia in palliative patients to be a result of chronic inflammation. Other causes like iron, folic acid and vitamin 12 deficiencies, parasitic infection, acute blood loss were not considered. This could be a limitation of the study. We only took blood transfusion as a reference over ESA. Further studies could be done comparing the beneficial effects of transfusion and ESA. Apart from symptoms of fatigue and breathlessness, other symptoms can also be considered for future research. In this study we did not take into account the diagnosis of the disease, the tumor site, size and grading of the disease. Better results can be obtained by classifying the tumor site and studying beneficial effects of transfusion on different types of cancer patients. Due to the lack of laboratory facilities and cost factor, this study also failed to measure the level of cytokines which is one important factor causing anemia in cancer. Also, we did not consider the chemotherapy regimen used for cancer treatment and its probable effect in anemia was also ignored.

In spite of being a crucial piece of the thread, we failed to report the transfusion reactions in our study. Though no late transfusion reactions were observed, some early transfusion reactions like thrombophlebitis and non-hemolytic febrile reactions was noted.

## Conclusion

Anemia is present in a majority of cancer palliative patients. Symptomatic benefits to CRF and breathlessness secondary to anemia can be achieved by packed red cell transfusion; however transfusion should be customized to an individual patient by an experienced palliative care physician in accordance with patients’ preferences. The authors recommend not withholding packed cell transfusion in cancer palliative patients as the study reported no increased risks of adverse events post-transfusion. Larger population studies are to be conducted before arriving to any conclusions.

## Data Availability

The datasets obtained and/or analyzed during the current study are not publicly available due to confidentiality consent of the study but can be obtained from the corresponding author on reasonable request.

## Abbreviations

CIA: Cancer Induced Anemia
CRF: Cancer Related Fatigue
DI: Dyspnea Index
ESA: Erythropoiesis Stimulating Agents
Hb: Hemoglobin
NCCN: National Comprehensive Cancer Network [26]
PS: Performance Status
QoL: Quality of Life
RBC: Red Blood Cells
WHO: World Health Organization

## References

[1] Torre Lindsey A., Bray Freddie, Siegel Rebecca L., Ferlay Jacques, Lortet-Tieulent Joannie, Jemal Ahmedin (2015). Global cancer statistics, 2012. CA: A Cancer Journal for Clinicians.

[2] Health Statistics and Information Systems:WHO Mortality Database [https://www.who.int/news-room/fact-sheets/detail/cancer].

[3] Definition of palliative care [http://www.who.int/cancer/palliative/definition/en/].

[4] Gautam P (2006). Palliative care services in Nepal. Kathmandu Univ Med J (KUMJ).

[5] Dicato M (2003). Anemia in cancer: some pathophysiological aspects. Oncologist.

[6] Monti Massimo, Castellani Lucia, Berlusconi Aurelia, Cunietti Ettore (1996). Use of red blood cell transfusions in terminally ill cancer patients admitted to a palliative care unit. Journal of Pain and Symptom Management.

[7] Calabrich A, Katz A (2011). Management of anemia in cancer patients. Future Oncol.

[8] Schrijvers D (2011). Management of anemia in cancer patients: transfusions. Oncologist.

[9] Smith LB, Cooling L, Davenport R (2013). How do I allocate blood products at the end of life? An ethical analysis with suggested guidelines. Transfusion (Paris).

[10] Niscola P, Tendas A, Scaramucci L, Giovannini M (2013). End of life care in hematology: still a challenging concern. Annals of Palliative Medicine.

[11] Liumbruno G, Bennardello F, Lattanzio A, Piccoli P, Rossetti G (2009). Recommendations for the transfusion of red blood cells. Blood Transfus.

[12] Douglas SP, Crook ED, Kirchner KA, Reynolds MD, Robinson CG (2001). “There is power in the blood”: a case discussing ethical issues of utility of resources. Am J Med Sci.

[13] Murphree DH, Kinard TN, Khera N, Storlie CB, Ngufor C, Upadhyaya S, Pathak J, Fortune E, Jacob EK, Carter RE (2017). Measuring the impact of ambulatory red blood cell transfusion on home functional status: study protocol for a pilot randomized controlled trial. Trials.

[14] Effect of Red Blood cell Transfusions on Patients- Reported Outcomes in an Ambulatory Oncology Population [https://www.firstwordpharma.com/node/1415812].

[15] Hébert PC, Wells G, Blajchman MA, Marshall J, Martin C, Pagliarello G, Tweeddale M, Schweitzer I, Yetisir E (1999). Group tTRiCCIftCCCT: a multicenter, randomized, controlled clinical trial of transfusion requirements in critical care. N Engl J Med.

[16] Preston NJ, Hurlow A, Brine J, Bennett MI (2012). Blood transfusions for anaemia in patients with advanced cancer. Cochrane Database Syst Rev.

[17] Gleeson C, Spencer D (1995). Blood transfusion and its benefits in palliative care. Palliat Med.

[18] Mock V, Atkinson A, Barsevick AM, Berger AM, Cimprich B, Eisenberger MA, Hinds P, Kaldor P, Otis-Green SA, Piper BF (2007). Cancer-related fatigue. Clinical practice guidelines in oncology. J Natl Compr Cancer Netw.

[19] Berger AM, Gerber LH, Mayer DK (2012). Cancer-related fatigue: implications for breast cancer survivors. Cancer.

[20] Maccio A, Madeddu C, Gramignano G, Mulas C, Tanca L, Cherchi MC, Floris C, Omoto I, Barracca A, Ganz T (2015). The role of inflammation, iron, and nutritional status in cancer-related anemia: results of a large, prospective, observational study. Haematologica.

[21] Ludwig Heinz, Van Belle Simon, Barrett-Lee Peter, Birgegård Gunnar, Bokemeyer Carsten, Gascón Pere, Kosmidis Paris, Krzakowski Maciej, Nortier Johan, Olmi Patrizia, Schneider Maurice, Schrijvers Dirk (2004). The European Cancer Anaemia Survey (ECAS): A large, multinational, prospective survey defining the prevalence, incidence, and treatment of anaemia in cancer patients. European Journal of Cancer.

[22] Monti M, Castellani L, Berlusconi A, Cunietti E (1996). Use of red blood cell transfusions in terminally ill cancer patients admitted to a palliative care unit. J Pain Symptom Manag.

[23] Dasch B, Kalies H, Feddersen B, Ruderer C, Hiddemann W, Bausewein C (2017). Care of cancer patients at the end of life in a German university hospital: a retrospective observational study from 2014. PLoS One.

[24] Hayward H, Hiersche A, Watson L (2012). Blood, glorious blood: are attitudes to blood transfusions for palliative purposes always positive?. BMJ Supportive &ampPalliative Care.

[25] Wachtel TJ, Mor V (1985). The use of transfusion in terminal cancer patients. Hospice versus conventional care setting. Transfusion.

[26] NCCN Guideline on Cancer- and Chemotherapy - Induced Anemia, Version 2.2018 [https://www.nccn.org/professionals/physician_gls/pdf/growthfactors.pdf].

[27] Littlewood TJ, Bajetta E, Nortier JW, Vercammen E, Rapoport B (2001). Effects of epoetin alfa on hematologic parameters and quality of life in cancer patients receiving nonplatinum chemotherapy: results of a randomized, double-blind, placebo-controlled trial. J Clin Oncol.

[28] Chin-Yee N, Taylor J, Rourke K, Faig D, Davis A, Fergusson D, Saidenberg E. Red blood cell transfusion in adult palliative care: a systematic review. Transfusion (Paris). 2017. 10.1111/trf.14413 29194669

[29] Sharma S, Sharma P, Tyler LN (2011). Transfusion of blood and products: indication and complications. Am Fam Physician.

[30] Eagleton HJ, Littlewood TJ (2003). Update on the clinical use and misuse of erythropoietin. Curr Hematol Malig Rep.

[31] Mercadante S, Ferrera P, Villari P, David F, Giarratano A, Riina S (2009). Effects of red blood cell transfusion on anemia-related symptoms in patients with cancer. J Palliat Med.

[32] Cella D (1997). The functional assessment of Cancer therapy-Anemia (FACT-an) scale: a new tool for the assessment of outcomes in cancer anemia and fatigue. Semin Hematol.

[33] Brown E, Hurlow A, Rahman A, Closs SJ, Bennett MI (2010). Assessment of fatigue after blood transfusion in palliative care patients: a feasibility study. J Palliat Med.

[34] LeBlanc TW, Eastman P, Neoh K, Agar MR, Rowett D, Vandersman Z, Currow DC, To THM, To LB (2017). The prospective evaluation of the net effect of red blood cell transfusions in routine provision of palliative care. J Palliat Med.

[35] Currow DC, To TH, To LB (2016). Can we detect transfusion benefits in palliative care patients?. J Palliat Med.

[36] Goksu SS, Gunduz S, Unal D, Uysal M, Arslan D, Tatli AM, Bozcuk H, Ozdogan M, Coskun HS (2014). Use of blood transfusion at the end of life: does it have any effects on survival of Cancer patients?. Asian Pac J Cancer Prev.

